# Willingness to share information on social media: a systematic literature review (2020–2024)

**DOI:** 10.3389/fpsyg.2025.1567506

**Published:** 2025-06-04

**Authors:** Yingying Cai, Syafila Kamarudin, Saiful Nujaimi

**Affiliations:** ^1^Department of Communication, Universiti Putra Malaysia, Serdang, Malaysia; ^2^Institute for Social Science Studies, Universiti Putra Malaysia, Serdang, Malaysia

**Keywords:** information sharing, social media, systematic literature review, misinformation, health information

## Abstract

**Introduction:**

The rapid advancement of Web 2.0 technologies, accelerated by the COVID-19 pandemic, has fundamentally transformed information sharing behaviors on social media. This transformation necessitates a comprehensive understanding of the factors influencing information sharing willingness in the digital era.

**Methods:**

This study systematically reviews 66 peer-reviewed journal articles published between 2020 and 2024, focusing on key research topics, theoretical frameworks, and methodologies used to examine information sharing willingness on social media. Following the PRISMA guidelines, articles were identified and analyzed using keyword matching, thematic categorization, and expert review.

**Results:**

Our findings reveal four core research themes: general information sharing, health-related information sharing, false information dissemination, and crisis information sharing. These themes are examined through three primary theoretical perspectives: motivational-driven theories, cognitive-processing theories, and social-relational theories. The study identifies key factors influencing information sharing willingness across motivational, cognitive, and social dimensions. Methodologically, survey-based studies dominate this field, with experimental designs providing supplementary insights.

**Discussion:**

This review contributes to the literature by providing a holistic synthesis of the current research landscape, identifying gaps in knowledge, and proposing potential directions for social media platform operators and policymakers to consider. Future research directions are proposed to address unresolved challenges and advance the theoretical and methodological understanding of information sharing behavior in the digital era.

## 1 Introduction

Social media platforms have become integral to modern communication, enabling connections through shared interests and facilitating real-time content dissemination. These digital environments support a spectrum of interactive behaviors, from personal status updates to reciprocal support exchanges, which collectively contribute to dynamic communication networks (Kapoor et al., 2018). Through the deliberate design of engagement mechanisms—including posting, commenting, and sharing functionalities—these platforms simultaneously strengthen interpersonal connections and illuminate the complex interrelationships among individuals, groups, and communities (Azzaakiyyah, 2023). This widespread adoption of social media, now encompassing over 5 billion active users globally (Petrosyan, 2024), reflecting a fundamental transformation in communication patterns. Platforms such as Facebook, Twitter, and Instagram illustrate this transformation, having significantly altered interpersonal interactions while also reconfiguring the relationship dynamics between businesses, organizations, and their respective audiences (Okonkwo et al., 2023).

For organizations, social media is utilized as a critical resource for gathering stakeholder feedback, while users frequently share reviews and opinions about products, services, and experiences. During the COVID-19 pandemic, governments and policymakers utilized social media platforms to distribute essential information to the public, underscoring their utility in public communication during emergencies (Ibrahim et al., 2024). However, the rapid dissemination of information on these platforms facilitates the propagation of unverified or inaccurate content, raising concerns about the authenticity, credibility, and potential biases of shared information (Adebesin et al., 2023). The circulation of misinformation and disinformation during crises, such as pandemics, is recognized for inciting public panic and diminishing trust in governing authorities. In light of these concerns, understanding the mechanisms, drivers, and outcomes of information sharing on social media emerges as a critical research priority, particularly in sensitive contexts such as public health, political communication, and knowledge management.

While existing reviews have provided valuable insights into context-specific aspects of information sharing, such as health-related information sharing (Benevento et al., 2023; Le et al., 2023; Naeem et al., 2022; van der Boon et al., 2024), cybersecurity concerns (Pala and Zhuang, 2019), and the spread of misinformation (Globig and Sharot, 2024), a comprehensive synthesis of overarching trends and theoretical frameworks remains lacking. Prior studies, such as the bibliometric analysis conducted by Abbas et al. (2022), have mapped the research landscape of information sharing. However, their review does not provide a systematic evaluation of the factors influencing individuals’ willingness to share information or the methodologies employed in studying this phenomenon.

To address this research need, a systematic review of the literature on information-sharing willingness in social media contexts is undertaken. Specifically, the study seeks to synthesize existing findings, identify emerging topics, and evaluate the methodologies, theoretical frameworks, and influencing factors explored in prior research. This review is guided by four key questions:

1. What are the main research themes addressed in the literature on information sharing willingness?
2. What theories are applied in the literature on information sharing willingness?
3. What factors influence individuals’ willingness to share information?
4. What research methods are employed in studying information sharing willingness?

By answering these questions, the study is intended to develop a comprehensive understanding of the field while providing a foundation for future research. The article is structured to introduce the topic (Section 1), followed by a description of the literature collection and screening process (Section 2), and then presents an analysis of key findings (Section 3). The concluding sections (Sections 4–7) provide a summary, recommendations for future research, discuss the research contributions, and address limitations.

## 2 Systematic literature review method

### 2.1 Article identification and selection

Drawing upon the SPIDER framework (Cooke et al., 2012), we developed a systematic and comprehensive search strategy. This framework was specifically chosen as it is particularly suitable for reviews of qualitative and mixed-methods research in social science contexts. Within this framework, we constructed our search strategy to target studies involving social media users as the sample (S), examining information sharing as the phenomenon of interest (PI), and focusing on willingness and intentions as the evaluation aspects (E). Design (D) and Research type (R) elements were intentionally kept broad in our search terms and implicitly incorporated through our inclusion criteria to ensure comprehensive capture of relevant methodological approaches.

For the search strategy, we utilized the following terms: ((willing* OR intention*) AND (“information shar*” OR “information disseminat*” OR “information spread*” OR “information forward*” OR “information disclos*” OR “content shar*”) AND (online* OR “social media” OR “social network*” OR internet* OR digital* OR web* OR “new media” OR virtual* OR platform* OR cyber*)). This approach ensured comprehensive capture of relevant studies while maintaining sufficient specificity to effectively identify literature concerning information sharing willingness among social media users.

To retrieve relevant research articles, we conducted a systematic search of the literature using three major databases: PubMed, Web of Science, and Scopus, which are widely recognized for their comprehensive coverage of relevant research. The search was restricted to articles published within the last 5 years, starting from 2020. The decision to focus on literature from 2020 onward is grounded in the significant contextual shift brought about by the COVID-19 pandemic, which emerged in late 2019 and escalated in 2020. This period highlighted the critical importance of information sharing in areas such as health communication, misinformation management, and crisis response. A total of 1,452 articles in English language were initially identified (PubMed: 202, Web of Science: 592, Scopus: 658) on 18 October 2024. After removing 479 duplicates using Microsoft Excel, 973 articles remained. Subsequently, 3 retracted publications were excluded. As a result, 970 articles were retained for the next phase of screening and eligibility assessment.

### 2.2 Inclusion/exclusion criteria

Article identification and selection followed the PRISMA guideline (Page et al., 2021), which provides a standardized framework for transparent and systematic reporting. Building on the SPIDER framework outlined in section 2.1, we developed specific inclusion and exclusion criteria that corresponded to each element of the framework (Sample, Phenomenon of Interest, Design, Evaluation, and Research type). Specifically, three criteria for inclusion were applied: (i) focus on the individual user perspective, or information was shared by individual users, (ii) focus on the information sharing or forwarding not disclosing, and (iii) take place in the context of social media platforms.

**Figure 1:**
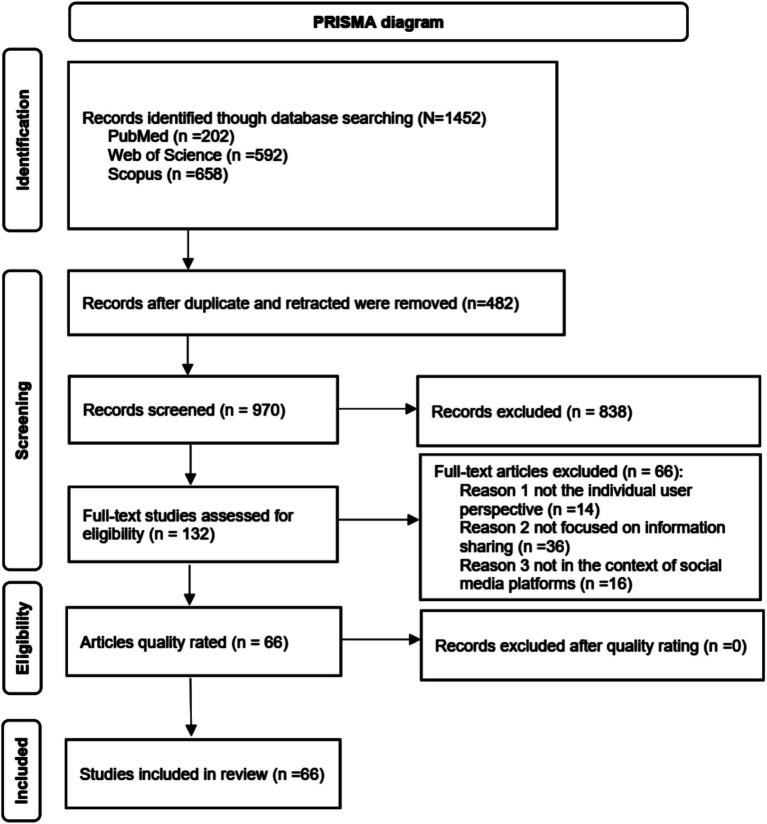
Article selection process.

Two reviewers screened the available abstracts and titles for relevance independently. After the initial screening by the first reviewer, a second reviewer independently verified the inclusion and exclusion decisions to ensure consistency. The flow diagram is presented in Figure 1 for reference. Based on these criteria, 132 articles were selected for full-text screening. Subsequently, only 66 articles proceeded to the quality assessment stage.

### 2.3 Quality assessment

We assessed the content validity of 66 articles using the criteria established by Hawker et al. (2002), applicable to both quantitative and qualitative studies. The evaluation process included nine sections: title and abstract, introduction and aims, methodology and data, sampling, analysis, ethical considerations, results, transferability, and usefulness. Each section was rated on a four-point scale, ranging from “good” (4 points) to “very poor” (1 point), with a total possible score of 36 points and a minimum threshold of 9 points. After the first reviewer independently evaluated the articles, the second reviewer examined the first reviewer’s evaluations. In cases of score discrepancies, a discussion was held to reach a consensus. Articles scoring between 26 and 34 points met the quality selection criteria (18–36 points). The final selected studies are listed in Appendix A, and the quality assessment table is presented in Appendix B.

### 2.4 Analysis techniques

To address the research questions, a combination of keyword matching, thematic analysis, and expert review was employed to ensure a comprehensive and reliable synthesis of the existing literature. The coding analysis was independently performed by two researchers, and the generated themes were reviewed by a third researcher to minimize subjectivity and ensure consistency.

For RQ1 – What are the main research themes addressed in the literature on information sharing willingness? – keywords, abstracts, and objectives of all selected articles were systematically analyzed. A codebook was created to map subtopics and their associated keywords, facilitating a comprehensive examination of full-text articles and classification of studies into specific domains.

For RQ2 – What theories are applied in literature on information sharing willingness? – the applied theoretical frameworks in the selected articles were identified and assessed. These frameworks, used to explore information sharing willingness, were coded and categorized into three primary groups: motivational-driven theories, cognitive-processing theories, and social-relational theories.

For RQ3 – What factors influence individuals’ willingness to share information? – the factors examined in the selected studies were extracted, coded based on their definitions, applications, and contexts, and subsequently grouped into three core dimensions: motivational factors, cognitive factors, and social factors.

For RQ4 – What research methods are employed in the study of information sharing willingness? – data collection techniques, research designs, and methods employed in the studies were analyzed. The findings were contextualized to emphasize the suitability of different methods for specific research objectives and to identify areas where methodological improvements could enhance the reliability and applicability of future studies.

## 3 Results and discussion

This section presents the key findings related to four primary research questions. First, key themes were identified in the literature on information sharing willingness. Second, the theories underpinning information sharing willingness were extracted and categorized into three types. Third, the analysis explores the factors influencing information sharing willingness, offering an overview of motivational, cognitive, and social factors. Finally, a review of the research methods employed in prior studies is provided, with particular attention to data collection techniques and their associated methodological limitations that remain unaddressed.

### 3.1 Research question 1: what are the main research themes addressed in the literature on information sharing willingness?

Table 1 provides a summary of the research topics examined in the literature on information-sharing willingness, addressing RQ1. Four major themes emerge as the most frequently studied: (i) general information sharing, represented by 26 articles; (ii) health-related information sharing, covered in 17 articles; (iii) false information dissemination, with 11 articles; and (iv) crisis information sharing, discussed in 8 articles.

**Table 1:** Summary of research topics on information sharing willingness.

Research topic	Number of studies	Focus areas
General information sharing	26	Broad exploration of information-sharing behaviors without restricting to specific platforms or information types.
Health-related information sharing	17	Sub-topics include:
		-General health information
		- Vaccine-related information
		- Health articles
		- COVID-19 information
False information dissemination	11	Sub-topics include:
		- Misinformation
		- False news
		- Health-related misinformation
Crisis information sharing	8	Sub-topics include:
		- Climate change risks
		- Disaster-related information
Crowdfunding information sharing	2	Motivations for sharing crowdfunding campaigns
Environmental information sharing	1	Focused on sharing environmental awareness content
Charitable information sharing	1	Examines willingness to share donation-related information

The review reveals that general information sharing and health-related information sharing are the most extensively studied topics, accounting for a combined total of 43 studies while emerging areas such as crowdfunding, environmental, and charitable information sharing remain significantly underexplored. This study corroborates the findings from Abbas et al. (2022), with the primary articles focusing on information sharing via social media, while identifying healthcare and COVID-19 are identified as trending topics. As Benevento et al. (2023) observed, the COVID-19 pandemic significantly increased patients’ willingness to share health information, particularly with healthcare institutions and researchers. This shift can likely be attributed to the immense burden posed by the pandemic, which also brought greater focus and urgency to the field of health information sharing.

These diverse research themes reflect the growing scholarly interest in understanding information sharing behaviors across various contexts, with significant attention given to general and health-related information sharing, while several specialized domains remain underexplored.

### 3.2 Research question 2: what theories are applied in the literature on information sharing willingness?

The theoretical frameworks used to explore information-sharing willingness can be categorized into three key groups: motivational-driven theories, cognitive-processing theories, and social-relational theories. Motivational-driven theories focus on the internal and external factors that drive individuals to share information. These theories emphasize personal attitudes, social norms, moral obligations, and individual gratifications as key determinants of behavior. Cognitive-processing theories focus on how individuals evaluate, interpret, and process information before deciding to share it. These theories consider the role of mental frameworks, message characteristics, and technological affordances in influencing sharing willingness. Social-relational theories examine how interpersonal relationships, social networks, and trust dynamics influence information-sharing behavior. These theories highlight the social context in which sharing occurs, focusing on the roles of relationships, community norms, and perceived influence. Table 2 presents the categorization of theories along with their corresponding frequencies.

**Table 2:** Classification of theories in information sharing willingness literature.

Category	Theories	Sources
Motivational-driven	Theory of planned behavior (11)	Ahmad et al. (2023), Alasmari and Zavalina (2022), Alwreikat (2021), Baah-Peprah et al. (2024), Chen (2020), Hong et al. (2021), Islam et al. (2020), Malik et al. (2021), Noh (2021), Wu and Kuang (2021), and Xiang et al. (2023)
	Uses and gratifications theory (8)	Chen (2020), Hong et al. (2021), Islam et al. (2020), Kim et al. (2021), Luo et al. (2023), Malik et al. (2021), Toh and Lee (2022), and Wu and Kuang (2021)
	Theory of reasoned action (5)	Gupta et al. (2021), Kim et al. (2020), Lin and Wang (2020), Tseng (2023), and Yossatorn et al. (2023)
	Health belief model (2)	Malik et al. (2022) and Shang et al. (2020)
	Extension of the extended parallel process model (2)	Lee (2020) and So et al. (2023)
	Norm activation model (1)	Chen (2020)
	Motivational theory (1)	Le (2022)
	Prototype willingness model (1)	Zhang and Cheng (2024)
	Self-presentation theory (1)	Zhang et al. (2021a)
	Impression management (1)	Hodson et al. (2022)
	Sociometer theory (1)	Lyngdoh et al. (2022)
	Flow theory (1)	Shree (2024)
	Hedonic motivation system acceptance model (1)	Shree (2024)
	Total: 36	
Cognitive-processing	Elaboration likelihood model (4)	Chen et al. (2021), Liu et al. (2024), Shang et al. (2020), and Zhang et al. (2021b)
	Technology acceptance model (3)	Shi et al. (2024), Shree (2024), and Xiang et al. (2023)
	Stimulus-organism-response model (3)	Chen et al. (2021), Guo et al. (2023), and Tang et al. (2024)
	information adoption model (1)	Li et al. (2022)
	Risk information seeking and sharing model (1)	Kim and Lai (2020)
	Cognitive appraisal theory (1)	Lu et al. (2021)
	Schema theory (1)	Liu et al. (2024)
	Dual-process theories of persuasion (1)	Gupta et al. (2021)
	Knowledge-attitude-practice theory (1)	Xiang et al. (2023)
	Attribution theory (1)	Pei et al. (2022)
	Attitude change model (1)	Gupta et al. (2021)
	Commitment-trust theory (1)	Kim et al. (2021)
	Total: 19	
Social-relational	Social capital theory (4)	Ahmad et al. (2023), Feng (2024), Hong et al. (2021), Noh (2021)
	Social cognitive theory (3)	Amriza et al. (2022), Ika Tamrin et al. (2021), and Wu and Kuang (2021)
	Trust theory (1)	Baah-Peprah et al. (2024)
	Signaling theory (1)	Baah-Peprah et al. (2024)
	Social amplification of risk framework (1)	Zhang et al. (2023)
	Influence of presumed influence model (1)	Paek et al. (2024)
	Reciprocity theories (1)	Li et al. (2022)
	Debt theories (1)	Li et al. (2022)
	Attachment theory (1)	Chou et al. (2022)
	Theory of interpersonal behavior (1)	Kim et al. (2019)
	Prosocial behavior theory (1)	Islam et al. (2020)
	Diffusion of innovations theory (1)	Alasmari and Zavalina (2022)
	The imagined audience (1)	Hodson et al. (2022)
	Social role theory (1)	Lin and Wang (2020)
	Total: 19	

In the field of information sharing, most studies adopt motivation-related theories (36), followed by cognitive-related theories (19) and social-related theories (19). These counts sum to 74, exceeding our sample of 66 studies because researchers often employ multiple theoretical frameworks simultaneously, particularly in survey-based research. The predominance of motivation-related theories aligns with Le et al. (2023) review of health information studies, which also emphasizes motivation has been widely recognized and extensively studied in health information sharing. Among the most frequently applied theories in this review are the Theory of Planned Behavior, Uses and Gratifications Theory, Theory of Reasoned Action. When categorized by theoretical dimensions, the Theory of Planned Behavior, Elaboration Likelihood Model, and Social Capital Theory emerge as the leading frameworks in the motivational, cognitive, and social dimensions, respectively.

### 3.3 Research question 3: what factors influence individuals’ willingness to share information?

These factors were categorized into three core dimensions: motivational factors, which address why individuals share information; cognitive factors, focusing on how individuals evaluate and process information; and social factors, exploring the relational and normative dynamics that shape sharing behavior.

#### 3.3.1 Motivational factors

Motivational factors explain why individuals choose to share information, focusing on intrinsic and extrinsic drivers. Gratification factors, including informational, entertaining, remunerative, and relational motives (Kim et al., 2021) and outcome expectations, such as information seeking, emotion regulation, altruism, and public engagement ^59^, play a crucial role in influencing users’ sharing willingness. Emotional states like anxiety further increase sharing willingness by enhancing self-efficacy and stimulating information-seeking behavior (Lee, 2020). Proactive individuals with high self-efficacy and who feel responsible for constructive change are more inclined to share even negative information, underscoring the role of agency in sharing decisions (Marler et al., 2021). In specific contexts, such as environmental information-sharing on platforms like WeChat, egoistic motives (e.g., self-presentation, socializing) and altruistic factors (e.g., awareness of consequences, responsibility attribution) interact with attitudes, norms, and behavioral willingness to shape actual sharing behavior (Chen, 2020). Additionally, people motivated by self-image goals are more likely to share self-promoting content than prosocial content, illustrating how personal goals influence sharing behaviors (Toh and Lee, 2022). Similarly, intrinsic motivations, such as emotional and informative appeals, and extrinsic drivers like perceived herding and crowd effects, strongly promote willingness and sharing (Le, 2022). However, Lv et al. (2022) noted that habitual behaviors, act as a bridge, mediated between motivations and sharing willingness.

#### 3.3.2 Cognitive factors

Cognitive factors emphasize how individuals evaluate and process information to decide whether to share it. Among these, perceived usefulness is a critical determinant, mediating the effects of argument quality and source credibility on health information-sharing willingness (Shang et al., 2020). Empirical evidence indicates that perceived usefulness derived from entertainment motives has a stronger influence on social media sharing than perceived usefulness derived from information-seeking motives, reflecting the contextual variability of sharing behaviors (Shree, 2024). Information quality and content valence significantly shape sharing willingness, with self-referential processing acting as a mediator between emotional content and sharing behavior, highlighting the importance of personal relevance in decision-making (Liu et al., 2024). Emotional and cognitive interactions further enhance sharing behavior. Specific emotional states such as anger and anxiety significantly shape sharing willingness, with individuals experiencing both emotions simultaneously exhibiting the highest likelihood of sharing emotionally charged content (Han et al., 2023). For instance, negative emotional appeals paired with pseudo-authoritative sources increase the perceived credibility of misinformation, thereby boosting sharing willingness; however, excessive negative appeals induce vigilant verification behavior, reducing sharing (Xue et al., 2024). Contextual factors also play a crucial role, with visual design and information overload moderating the effects of gratification factors on sharing willingness (Kim et al., 2019). Enhanced transparency in visual displays has been shown to improve willingness to share vaccination information by making critical details more comprehensible (Giese et al., 2021). Moreover, accuracy-nudge labels significantly reduce users’ misguided trust in misinformation, promote verification behaviors, and ultimately decrease misinformation sharing (Xue et al., 2024).

#### 3.3.3 Social factors

Social factors explore with whom and under what social conditions information is shared, emphasizing interpersonal relationships, norms, and trust. Bonding and bridging social capital serve as mediators between social connections and sharing willingness, reflecting the importance of network structure in shaping behavior (Ahmad et al., 2023). Subjective norms, attitudes, and social presence on social networking sites positively influence information-sharing willingness, supported by situational normality and structural assurance that foster trust in online environments (Tseng, 2023). Social media use indirectly influences corrective information-sharing willingness through trust, shared experiences, and social interaction connections, with health literacy moderating these effects (Feng, 2024). Individual social circumstances can further shape specific sharing patterns. For example, socially isolated individuals, particularly Gen Z users, are more likely to share personal information when experiencing fear of missing out or repetitive negative thoughts (Lyngdoh et al., 2022). Additionally, cultural norms and trust in online spaces shape a sharing culture that fosters collective responsibility and engagement (Noh, 2021).

Compared with the review of Naeem et al. (2022), who emphasized trust as a key determinant in health information sharing, our study extends this knowledge by examining trust within social media contexts. Naeem et al. (2022) focused primarily on healthcare settings, whereas our research investigates information sharing dynamics across digital platforms. This extension is significant as Hashim et al. (2020) found that during the COVID-19 pandemic, social media was the most popular information channel despite television being most trusted. Our study further contributes by identifying institutional trust hierarchies, where Lu et al. (2021) found healthcare professionals, academic institutions, and government agencies serve as trusted information sources with specific sharing motivations. Additionally, we analyze bidirectional trust (in sources and recipients) affecting information sharing attitudes (Zhang et al., 2021b), and identify three trust-building pathways in online information dissemination: content quality, initiator credibility, and platform reputation (Chen et al., 2021). These contributions advance our understanding of information sharing behavior beyond existing healthcare-focused reviews.

### 3.4 Research question 4: what research methods are employed in studying information sharing willingness?

As summarized in Table 3, which provides an overview of the research methods and focus areas explored in these studies, survey methods—comprising 42 out of the 66 articles reviewed—emerge as the predominant research approach for examining information sharing behaviors. These studies are primarily concentrated in three key research contexts: general information sharing (18 articles), health information-sharing willingness (13 articles), and crisis information sharing (6 articles). Additionally, smaller subsets of studies focus on misinformation sharing (3 articles), crowdfunding information sharing (1 article), and environmental information sharing (1 article). These studies, while contributing valuable insights into information sharing, have several methodological limitations that warrant attention in future research. Many studies rely heavily on self-reported data, which introduces potential biases and limits the ability to establish causal relationships due to the predominance of cross-sectional designs (Ika Tamrin et al., 2021; Lee, 2020; Tang et al., 2024; Yossatorn et al., 2023). Sampling issues, including the use of convenience samples (Kim et al., 2019), over-reliance on specific platforms (e.g., Facebook or WeChat) (Lin and Wang, 2020; Sun et al., 2021; Tseng, 2023), and a focus on specific demographic groups (e.g., university students or culturally homogenous populations) (Ahmad et al., 2023; Yossatorn et al., 2023), restrict the generalizability of findings to broader and more diverse populations. Platform-specific characteristics and the variability in ease of use across social media platforms are also rarely addressed, limiting insights into cross-platform behavior (Shree, 2024).

**Table 3:** Overview of research methods and focus areas in information sharing studies.

Research method	Focus areas	Limitation
Survey methods (42)	-General information sharing (18 articles) - Health information sharing (13 articles) - Crisis information sharing (6 articles) - Misinformation sharing (3 articles) - Crowdfunding information sharing (1 article) -Environmental information sharing (1 article)	- Heavy reliance on self-reported data introduces biases and limits causal inference due to cross-sectional designs (Ika Tamrin et al., 2021; Lee, 2020). - Sampling issues: convenience samples, platform-specific studies (e.g., Facebook, WeChat), and culturally homogenous demographics limit generalizability (Kim et al., 2019; Ahmad et al., 2023). - Lack of longitudinal designs limits understanding of temporal dynamics (Tang et al., 2024).
Experimental methods (21)	- Misinformation sharing (8 articles) -General information sharing (8 articles) - Health information sharing (3 articles) -Charitable information sharing (1 article) - Crisis information sharing (1 article)	- Non-representative samples (e.g., university students, convenience samples) reduce generalizability (Fazio, 2020; Zhang et al., 2021a). - Platform-specific focus restricts cross-platform comparisons (e.g., Telegram, WhatsApp) (Lu et al., 2022; Miri et al., 2024). - Simplified designs and hypothetical scenarios lack ecological validity (Pennycook et al., 2020). - Experiments often measure hypothetical willingness instead of actual behaviors (Chen et al., 2021).
Mixed methods (2)	- Crowdfunding information sharing (1 article) - Crisis information sharing (1 article)	- Highlighted inconsistencies between studies within the same research, raising concerns about reliability and generalizability (Pei et al., 2022).
Interviews (1)	- Health information sharing (1 article)	- Relied on self-selected, culturally homogenous participants (e.g., predominantly White, similar educational backgrounds), limiting generalizability (Hodson et al., 2022).

A total of 21 studies employed experimental methods, making it a significant approach in understanding information-sharing behaviors. The majority of these studies focused on misinformation sharing (8 articles) and general information sharing (8 articles). Additionally, health information sharing was explored in 3 studies, while charitable information sharing and crisis information sharing were the focus of 1 study each. Experimental studies on information-sharing behaviors provide valuable insights into causal relationships but face several methodological limitations that warrant attention. A significant challenge lies in the reliance on non-representative samples, such as convenience samples or specific demographic groups such as university students aged 18–24, which limits the generalizability of findings to broader populations (Fazio, 2020; Pennycook et al., 2020; Zhang, Li, et al., 2021). Many studies also focus on single social media platforms (e.g., Telegram, WhatsApp) (Lu et al., 2022; Miri et al., 2024), restricting cross-platform comparisons and failing to account for the diverse interaction mechanisms present across platforms like Twitter, Facebook, and Snapchat. Additionally, experiments often use simplified designs, such as single-message stimuli or hypothetical scenarios, which reduce ecological validity and fail to reflect the complexity of real-world information-sharing environments where users are exposed to conflicting or diverse content (Pennycook et al., 2020). Self-reported data further introduce potential biases, including social desirability and memory recall errors, which may not accurately represent actual sharing behaviors (Kim et al., 2020; Miri et al., 2024; Yang and Overton, 2022). Controlled laboratory experiments, while ensuring high internal validity, often face challenges in external validity, as findings may not generalize to naturalistic social media contexts (Suárez Vázquez and Chica Serrano, 2021; Xue et al., 2024; Zhao et al., 2020). Furthermore, many studies neglect to measure actual behaviors, such as the number of posts shared or donations made, focusing instead on hypothetical sharing willingness (Chen et al., 2021). This gap between willingness and behavior limits the practical applicability of findings (Xue et al., 2024; Yang and Overton, 2022).

A total of 2 articles employed mixed research methods, focusing on the contexts of crowdfunding information and crisis information sharing, respectively. One of these mixed-methods studies revealed inconsistencies between the context of Study 1 and those of Studies 2 and 3, raising concerns about the robustness, reliability, and generalizability of the research findings (Zhang et al., 2023). Additionally, one article utilized an online interview method, specifically investigating health information sharing. This interview-based study relied on a self-selected, culturally homogeneous sample that was predominantly White and had similar educational backgrounds. This sample composition limits the generalizability of the findings as it primarily reflects the needs and experiences of a specific demographic (Hodson et al., 2022).

The findings provide a detailed exploration of the methodological approaches used to study the information-sharing willingness, highlighting survey methods as the predominant approach and experimental methods as a critical means. Compared to Abbas et al. (2022), who focus on the evolution of studies in terms of authors, journals, citations, and also topics in social media-based sharing, this study emphasizes methodological limitations, such as over-reliance on self-reported data and cross-sectional designs, which restrict causal inferences and generalizability. This study, however, aligns with Le et al. (2023), confirming that surveys remain the predominant method used in studies on health information sharing. However, this study primarily measures the willingness to share information through surveys, and the interview method is not prominently featured in this study, which is contradicted with Benevento et al. (2023), who indicated that most studies on the willingness to share health-related information are conducted using surveys or interviews.

## 4 Directions for future research

The findings reveal that existing research has predominantly focused on themes such as general information sharing, health-related information sharing, the dissemination of false information, and information sharing during crises. However, comparatively less attention has been given to broader contexts, including environmental and crowdfunding information sharing. This emphasis on certain themes highlights a gap in the literature and underscores the necessity for future studies to explore a wider range of contexts, thereby enriching the comprehensiveness of the field.

Secondly, the analysis of theoretical frameworks reveals the dominance of motivational-driven theory, particularly the Theory of Planned Behavior and the Uses and Gratifications Theory. Existing frameworks, such as the Uses and Gratifications Theory, require expansion to incorporate constructs including habits (Malik et al., 2021), altruism, and trust (Lyngdoh et al., 2022), offering a more nuanced understanding of sharing behaviors. Emotional states, including anger, fear, and hope, also warrant deeper investigation, particularly in their temporal evolution and interplay with sharing willingness (Han et al., 2023; Lee, 2020). Furthermore, platform-specific features (e.g., Instagram, TikTok, Twitter) (Ika Tamrin et al., 2021; Kim et al., 2021; Miri et al., 2024; Shi et al., 2024; Tseng, 2023) and contextual factors, such as message framing (emotional vs. rational, gain vs. loss) (Miri et al., 2024; Shang et al., 2020), significantly shape user engagement. These areas remain underexplored, underscoring the need for tailored strategies that align with user behaviors across diverse platforms and thematic contexts.

Thirdly, survey methodologies emerged as the most frequently employed research approach, with a limited number of studies utilizing experimental designs or mixed methods. The reliance on cross-sectional studies limits the ability to establish causality, emphasizing the need for longitudinal insights (Tang et al., 2024). The integration of experimental designs with real-world data would further enhance external validity (Suárez Vázquez and Chica Serrano, 2021). Addressing sampling biases is equally critical, as current studies often neglect underrepresented groups, including the elderly, rural populations, and individuals with low digital literacy (Lu et al., 2021; Malik et al., 2022; Toh and Lee, 2022; Zhao et al., 2020). Employing multi-stage sampling techniques could improve the generalizability of findings (Tang et al., 2024). Advanced analytical methods, such as system dynamics modeling and behavioral observation, offer promising avenues for uncovering complex interactions between willingness and sharing behaviors (Shi et al., 2024). These advancements will enable a more inclusive, robust, and context-sensitive understanding of information-sharing willingness.

## 5 Research contributions

This study makes significant contributions to understanding information-sharing willingness in the digital age, particularly within diverse online contexts. Theoretically, it expands the knowledge of research distributions, frameworks, and methodologies, identifying critical gaps such as the limited inclusion of diverse demographics and insufficient exploration of cultural and contextual influences. It emphasizes the integration of emotional dynamics, platform-specific features, and message framing while advocating for the evolution of established frameworks like Uses and Gratifications Theory to encompass constructs such as trust, altruism, and habits. Practically, social media platforms and policymakers should develop interventions targeting the motivational, cognitive, and social factors that influence information-sharing behaviors, particularly in the areas of health communication and misinformation management.

## 6 Limitations

This study has several limitations that warrant attention. First, the research relies on data from only three databases, which may not comprehensively capture the breadth of relevant literature, thereby introducing potential biases stemming from database-specific inclusivity. Second, the study exclusively focuses on articles addressing information sharing willingness, omitting studies that examine information-sharing behaviors in isolation. This narrower scope, while purposeful, limits the ability to holistically understand the broader spectrum of information-sharing processes. Third, to ensure a focus on contemporary developments, the study includes only articles published within the past 5 years. While this approach highlights recent advancements, it may inadvertently exclude foundational studies and longitudinal trends that have shaped the evolution of the field. Future research could address these limitations by employing bibliometric analysis or meta-analytic approaches to synthesize findings across a broader temporal and methodological scope.

## 7 Conclusion

Following the PRISMA procedure, this study identified 66 journal articles examining information-sharing willingness. The analysis synthesizes the existing literature, categorizing research into four main topics: general information sharing, health-related information sharing, false information dissemination, and crisis information sharing. These studies are grounded in three primary theoretical frameworks: motivation-driven theories, cognitive-processing theories, and social-relational theories. The factors influencing information sharing behaviors are classified into motivational factors, cognitive factors, and social factors. Methodologically, survey-based studies dominate the field, complemented by experimental approaches.

## Data Availability

The original contributions presented in the study are included in the article/Supplementary material, further inquiries can be directed to the corresponding author.
