# Supplementary material for: Willingness to share information on social media: a systematic literature review (2020–2024)
Source: Front Psychol. 2025 Jun 4;16:1567506. doi: 10.3389/fpsyg.2025.1567506 (PMC12174407; doi:10.3389/fpsyg.2025.1567506)
Supplement: Supplementary file 2 [file Supplementary_file_2.docx]

| **Index** | **Title** | **Journal** | **Author** | **Method** | **theory** | **Country** | **Information Type** |
| --- | --- | --- | --- | --- | --- | --- | --- |
| 1 | “In this together”: on the antecedents and implications of crowdfunding community identification and trust | Venture Capital | (Baah-Peprah et al., 2024) | Survey | Trust theory；signaling theory；the theory of planned behavior | Finland | Crowdfunding Information |
| 2 | Research on user donation and information sharing in textile crowdfunding | Industria Textila | (Pei et al., 2022) | Mixed method | Attribution theory | China | Crowdfunding Information |
| 3 | Bonding and bridging social capital as predictors of information sharing intention and behavior among Pakistani Facebook users | Information Development | (Ahmad et al., 2023) | Survey | Social capital theory; theory of planned behavior | Pakistan | General Information |
| 4 | Can do and reason to: when are proactive employees willing to share negative information? | International Journal of Organizational Analysis | (Marler et al., 2021) | Survey | None | USA | General Information |
| 5 | Examining gender differences in people’s information-sharing decisions on social networking sites | International Journal of Information Management | (Lin & Wang, 2020) | Survey | Theory of reasoned action; Social role theory | USA | General Information |
| 6 | Exploring the effects of sense of membership on information sharing in virtual communities | Journal of Electronic Commerce Research | (Luo et al., 2023) | Survey | Uses and gratifications theory | China | General Information |
| 7 | Exploring the Information-Sharing Intention on Social Networking Sites | Asia Pacific Journal of Information Systems | (Tseng, 2023) | Survey | The theory of reasoned action | Taiwan, China | General Information |
| 8 | Gaming with health misinformation: a social capital-based study of corrective information sharing factors in social media | Frontiers in Public Health | (Feng, 2024) | Survey | Social capital theory | China | Health Information |
| 9 | How do users select the content they share on social media: flow theory perspective | Online Information Review | (Shree, 2024) | Survey | Technology acceptance model; Flow theory; Hedonic Motivation System Acceptance Model | India | General Information |
| 10 | How event information is trusted and shared on social media: a uses and gratification perspective | Journal of Travel & Tourism Marketing | (Kim et al., 2021) | Survey | Uses and gratifications theory; commitment-trust theory | USA | General Information |
| 11 | How the strength of social ties influences users’ information sharing and purchase intentions | Current Psychology | (Sun et al., 2021) | Survey | None | China | General Information |
| 12 | Information-Sharing Behaviors Among Sports Fans Using #Hashtags | Communication & Sport | (Kim et al., 2019) | Survey | Theory of interpersonal behavior | USA | General Information |
| 13 | Intention to share: the relationship between cybersecurity behaviour and sharing specific content in Facebook | Indonesian Journal of Science and Technology | (Ika Tamrin et al., 2021) | Survey | Social cognitive theory | Malaysia | General Information |
| 14 | Investigating the Relationships Among LINE Users’ Concerns, Motivations for Information Sharing Intention and Information Sharing Behavior | Sage Open | (Yossatorn et al., 2023) | Survey | The theory of reasoned action | Thailand | General Information |
| 15 | Is That Insta Worthy? Predicting Content Sharing Behavior on Social Media Through Interpersonal Goals | Cyberpsychology: Journal of Psychosocial Research on Cyberspace | (Toh & Lee, 2022) | Experiment | Uses and gratifications theory | USA | General Information |
| 16 | Message self and social relevance increases intentions to share content: Correlational and causal evidence from six studies | Journal of experimental psychology | (Cosme et al., 2023) | Experiment | None | USA | General Information |
| 17 | Others’ fortune in online vs offline settings: how envy affects people’s intention to share information | Internet Research | (Suárez Vázquez & Chica Serrano, 2021) | Experiment | None | N/A | General Information |
| 18 | Predicting intention to share information on commercial websites based on personality traits | The Bottom Line | (Soltani-Nejad et al., 2020) | Survey | None | Iran | General Information |
| 19 | Predictors of Online News-Sharing Intention in the U.S and South Korea: An Application of the Theory of Reasoned Action | Communication Studies | (Kim et al., 2020) | Experiment | The theory of reasoned action | USA and South Korea | General Information |
| 20 | Propagation of information sharing in social media: the perspective of intrinsic and extrinsic cues | VINE Journal of Information and Knowledge Management System | (Le, 2022) | Survey | Motivational theory | Vietnam | General Information |
| 21 | Public and Private Information Sharing under “New Normal” of COVID-19: Understanding the Roles of Habit and Outcome Expectation | International Journal of Environmental Research and Public Health | (Lv et al., 2022) | Survey | None | China | General Information |
| 22 | Social isolation and social anxiety as drivers of generation Z's willingness to share personal information on social media | Psychology & Marketing | (Lyngdoh et al., 2022) | Experiment | Sociometer theory | India | General Information |
| 23 | The secret to successful evocative messages: Anger takes the lead in information sharing over anxiety | Communication Monographs | (Han et al., 2023) | Experiment | None | South Korea | General Information |
| 24 | Understanding Social Media Information Sharing in Individuals with Depression: Insights from the Elaboration Likelihood Model and Schema Activation Theory | Psychology research and behavior management | (Liu et al., 2024) | Experiment | The elaboration likelihood model; Schema theory | China | General Information |
| 25 | Understanding viewers’ information-sharing in live-streaming based on a motivation perspective | Online Information Review | (Chou et al., 2022) | Survey | Attachment theory | Taiwan, China | General Information |
| 26 | Users’ unverified information-sharing behavior on social media: The role of reasoned and social reactive pathways | Acta Psychologica | (Zhang & Cheng, 2024) | Survey | Prototype willingness model | China | General Information |
| 27 | What type of purchase do you prefer to share on social networking sites: Experiential or material? | Journal of Retailing and Consumer Services | (Zhang, Li, et al., 2021) | Experiment | Self-presentation theory | China | General Information |
| 28 | When we seek and share information about mental illness: The impact of threat appraisal, negative emotions, and efficacy | The Social Science Journal | (Lee, 2020) | Survey | The extended parallel process model | USA | General Information |
| 29 | Why do We Share Information? Explaining Information Sharing Behavior through a New Conceptual Model between Sharer to Receiver within SNS | Asia Pacific Journal of Information Systems | (Noh, 2021) | Survey | Social capital theory; theory of planned behavior | N/A | General Information |
| 30 | Does disseminating (mis)information restore social connection during a global pandemic? | Social and Personality Psychology Compass | (Wicks et al., 2023) | Experiment | None | USA | False Information |
| 31 | Moral leniency towards belief-consistent disinformation may help explain its spread on social media | PLOS ONE | (Joyner et al., 2023) | Experiment | None | England | False Information |
| 32 | Pausing to consider why a headline is true or false can help reduce the sharing of false news | Harvard Kennedy School Misinformation Review | (Fazio, 2020) | Experiment | None | USA | False Information |
| 33 | Sharing of misinformation during COVID-19 pandemic: Applying the Theory of Planned Behavior with the integration of perceived severity. | Science & Technology Libraries | (Alwreikat, 2021) | Survey | Theory of planned behavior | Jordan | False Information |
| 34 | The impact of emotional vs rational message framing on social media users’ detection and sharing of misinformation: an experimental study | Journal of Information, Communication and Ethics in Society | (Miri et al., 2024) | Experiment | None | Iran | False Information |
| 35 | Think before you Share: Beliefs and emotions that shaped COVID-19 (Mis)information vetting and sharing intentions among WhatsApp users in the United Kingdom | Telematics and Informatics | (Lu et al., 2022) | Experiment | None | UK | False Information |
| 36 | What If Unmotivated Is More Dangerous? The Motivation-Contingent Effectiveness of Misinformation Correction on Social Media | International Journal of Communication | (Yang & Overton, 2022) | Experiment | None | USA | False Information |
| 37 | Diffusion of COVID-19 misinformation: Mechanisms for threat- and efficacy-related misinformation diffusion | Computers in Human Behavior | (So et al., 2023) | Survey | The extended parallel process model | South Korea | False Information |
| 38 | Fighting COVID-19 Misinformation on Social Media: Experimental Evidence for a Scalable Accuracy-Nudge Intervention | Psychological Science | (Pennycook et al., 2020) | Experiment | None | USA | False Information |
| 39 | Mitigating the influence of message features on health misinformation sharing intention in social media: Experimental evidence for accuracy-nudge intervention | Social Science & Medicine | (Xue et al., 2024) | Experiment | None | China | False Information |
| 40 | Understanding health misinformation sharing among the middle-aged or above in China: roles of social media health information seeking, misperceptions and information processing predispositions | Online Information Review | (Tang et al., 2024) | Survey | Stimulus-organism-response model | China | False Information |
| 41 | Differential effects of digital media platforms on climate change risk information-sharing intention: A moderated mediation model | Risk Analysis | (Paek et al., 2024) | Survey | The influence of presumed influence model | South Korea | Crisis Information |
| 42 | How does social presence influence public crisis information sharing intention? Situational pressure perspective | Frontiers in Public Health | (Guo et al., 2023) | Survey | Stimulus-organism-response model | China | Crisis Information |
| 43 | How risk messages influence tourist processing and sharing: The role of emojis | Journal of Hospitality and Tourism Management | (Zhang et al., 2023) | Mixed method | The Social Amplification of Risk Framework | China | Crisis Information |
| 44 | Seeking and Sharing Information about Transboundary Air Pollution in Singapore: Effects of Own and Others’ Information Insufficiency | Environmental Communication | (Kim & Lai, 2020) | Survey | Risk information seeking and sharing model | Singapore | Crisis Information |
| 45 | The Impact of Personal, Environmental, and Information Platform Factors on Disaster Information Sharing on Twitter | Register: Jurnal Ilmiah Teknologi Sistem Informasi | (Amriza et al., 2022) | Survey | Social cognitive theory | Indonesia | Crisis Information |
| 46 | The media risk of infodemic in public health emergencies: Consequences and mitigation approaches | PLoS One | (Shi et al., 2024) | Survey | The technology acceptance model | China | Crisis Information |
| 47 | Understanding social media users’ engagement intention toward emergency information: the role of experience and information usefulness in a reciprocity framework | Information Technology & People | (Li et al., 2022) | Survey | Reciprocity theory; indebtedness theory; information adoption model | China | Crisis Information |
| 48 | What motivates information sharing about disaster victims on social media? Exploring the role of compassion, sadness, expectancy violation, and enjoyment | International Journal of Disaster Risk Reduction, | (Lu & Yuan, 2021) | Experiment | None | USA | Crisis Information |
| 49 | Accuracy-sensitisation promotes the sharing of pro- (but not anti-) vaccine information | Psychology & health | (Saling et al., 2024) | Experiment | None | Australia | Health Information |
| 50 | Configuration Path Study of Influencing Factors on Health Information-Sharing Behavior among Users of Online Health Communities: Based on SEM and fsQCA Methods | Healthcare | (Xiang et al., 2023) | Survey | Theory of planned behavior; technology acceptance model; “knowledge-attitude-practice” theory | China | Health Information |
| 51 | Determinants of information diffusion in online communication on vaccination: The benefits of visual displays | Vaccine | (Giese et al., 2021) | Experiment | None | USA | Health Information |
| 52 | Exploring Influence Factors of WeChat Users’ Health Information Sharing Behavior: Based on an Integrated Model of TPB, UGT and SCT | International Journal of Human–Computer Interaction | (Wu & Kuang, 2021) | Survey | The theory of planned behavior; uses and gratifications; social cognitive theory | China | Health Information |
| 53 | Health information seeking and sharing behavior of young adults on social media in Pakistan | Journal of Librarianship and Information Science | (Malik et al., 2022) | Survey | Health belief model | Pakistan | Health Information |
| 54 | “My People Already Know That”: The Imagined Audience and COVID-19 Health Information Sharing Practices on Social Media | Social Media + Society | (Hodson et al., 2022) | Interviews | The imagined audience；Impression Management | Canada | Health Information |
| 55 | Promoting users’ intention to share online health articles on social media: The role of confirmation bias | Information Processing & Management | (Zhao et al., 2020) | Experiment | None | China | Health Information |
| 56 | Source Trust and COVID-19 Information Sharing: The Mediating Roles of Emotions and Beliefs About Sharing | Health Education & Behavior | (Lu et al., 2021) | Survey | Cognitive appraisal theory | China | Health Information |
| 57 | Students' Intention to Share Information Via Social Media: A Case Study of Covid-19 Pandemic | Indonesian Journal of Science and Technology | (Hossain Parash et al., 2020) | Survey | None | Malaysia | Health Information |
| 58 | The role of social influencers for effective public health communication | Online Information Review | (Gupta et al., 2021) | Survey | Attitude change model; dual-process theories of persuasion; the theory of reasoned action | India | Health Information |
| 59 | Understanding Knowledgeable Workers’ Behavior Toward COVID-19 Information Sharing Through WhatsApp in Pakistan | Frontiers in psychology | (Islam et al., 2020) | Survey | Uses and gratifications theory; theory of prosocial behavior; theory of planned behavior | Pakistan | Health Information |
| 60 | Understanding older adults’ intention to share health information on social media: the role of health belief and information processing | Internet Research | (Shang et al., 2020) | Survey | Health belief model; elaboration likelihood model | China | Health Information |
| 61 | Understanding the Facebook Users’ Behavior towards COVID-19 Information Sharing by Integrating the Theory of Planned Behavior and Gratifications | Information Development | (Malik et al., 2021) | Survey | Uses and gratifications theory; theory of planned behavior | Pakistan | Health Information |
| 62 | Understanding the Health Information Sharing Behavior of Social Media Users: An Empirical Study on WeChat | Journal of Organizational and End User Computing | (Hong et al., 2021) | Survey | The theory of planned behavior; Social capital; Uses and gratifications theory | China | Health Information |
| 63 | Users’ health information sharing intention in strong ties social media: context of emerging markets | Library Hi Tech | (Zhang, Lin, et al., 2021) | Survey | Elaboration likelihood model | China | Health Information |
| 64 | Using Two Theories in Exploration of the Health Information Diffusion on Social Media During a Global Health Crisis | Journal of Information & Knowledge Management | (Alasmari & Zavalina, 2022) | Survey | Theory of Planned Behavior; Diffusion of Innovation Theory | USA | Health Information |
| 65 | An Investigation of the Influencing Factors of Chinese WeChat Users’ Environmental Information-Sharing Behavior Based on an Integrated Model of UGT, NAM, and TPB | Sustainability | (Chen, 2020) | Survey | Uses and gratification theory; theory of planned behavior; norm activation model | China | Environmental Information |
| 66 | Understanding people’s participation in online charities: a dual-process approach of trust and empathic concern | Industrial Management & Data Systems | (Chen et al., 2021) | Experiment | The elaboration likelihood model; stimulus–organism–response model | China | Charitable Information |

Ahmad, Z., Soroya, S. H., & Mahmood, K. (2023). Bonding and bridging social capital as predictors of information sharing intention and behavior among Pakistani Facebook users. *Information Development*. <https://doi.org/10.1177/02666669231200458>

Alasmari, H., & Zavalina, O. L. (2022). Using Two Theories in Exploration of the Health Information Diffusion on Social Media During a Global Health Crisis. *Journal of Information & Knowledge Management*, *22*(02). <https://doi.org/10.1142/s0219649222500952>

Alwreikat, A. (2021). Sharing of Misinformation during COVID-19 Pandemic: Applying the Theory of Planned Behavior with the Integration of Perceived Severity. *Science & Technology Libraries*, *41*(2), 133-151. <https://doi.org/10.1080/0194262x.2021.1960241>

Amriza, R. N. S., Ngafidin, K. N. M., & Ratnasari, W. (2022). The Impact of Personal, Environmental, and Information Platform Factors on Disaster Information Sharing on Twitter. *Register: Jurnal Ilmiah Teknologi Sistem Informasi*, *8*(2), 104-121. <https://doi.org/10.26594/register.v8i2.2540>

Baah-Peprah, P., Shneor, R., & Munim, Z. H. (2024). “In this together”: on the antecedents and implications of crowdfunding community identification and trust. *Venture Capital*, 1-31. <https://doi.org/10.1080/13691066.2024.2310232>

Chen, H., Li, W., Lyu, T., & Zheng, X. (2021). Understanding people's participation in online charities: a dual-process approach of trust and empathic concern. *Industrial Management & Data Systems*, *121*(7), 1642-1663. <https://doi.org/10.1108/imds-09-2020-0513>

Chen, Y. (2020). An Investigation of the Influencing Factors of Chinese WeChat Users’ Environmental Information-Sharing Behavior Based on an Integrated Model of UGT, NAM, and TPB. *Sustainability*, *12*(7). <https://doi.org/10.3390/su12072710>

Chou, S.-W., Hsieh, M.-C., & Pan, H.-C. (2022). Understanding viewers' information-sharing in live-streaming based on a motivation perspective. *Online Information Review*, *47*(1), 177-196. <https://doi.org/10.1108/oir-12-2020-0576>

Cosme, D., Scholz, C., Chan, H. Y., Dore, B. P., Pandey, P., Carreras-Tartak, J.,…Falk, E. B. (2023). Message self and social relevance increases intentions to share content: Correlational and causal evidence from six studies. *J Exp Psychol Gen*, *152*(1), 253-267. <https://doi.org/10.1037/xge0001270>

Fazio, L. (2020). Pausing to consider why a headline is true or false can help reduce the sharing of false news. *Harvard Kennedy School Misinformation Review*. <https://doi.org/10.37016/mr-2020-009>

Feng, B. (2024). Gaming with health misinformation: a social capital-based study of corrective information sharing factors in social media. *Frontiers in Public Health*, *12*, 1351820. <https://doi.org/10.3389/fpubh.2024.1351820>

Giese, H., Neth, H., & Gaissmaier, W. (2021). Determinants of information diffusion in online communication on vaccination: The benefits of visual displays. *Vaccine*, *39*(43), 6407-6413. <https://doi.org/10.1016/j.vaccine.2021.09.016>

Guo, X., Jin, H., & Qi, T. (2023). How does social presence influence public crisis information sharing intention? Situational pressure perspective. *Front Public Health*, *11*, 1124876. <https://doi.org/10.3389/fpubh.2023.1124876>

Gupta, S., Dash, S. B., & Mahajan, R. (2021). The role of social influencers for effective public health communication. *Online Information Review*, *46*(5), 974-992. <https://doi.org/10.1108/oir-01-2021-0012>

Han, J., Lee, S. E., & Cha, M. (2023). The secret to successful evocative messages: Anger takes the lead in information sharing over anxiety. *Communication Monographs*, *90*(4), 545-565. <https://doi.org/10.1080/03637751.2023.2236183>

Hodson, J., O'Meara, V., Thompson, C., Houlden, S., Gosse, C., & Veletsianos, G. (2022). "My People Already Know That": The Imagined Audience and COVID-19 Health Information Sharing Practices on Social Media. *Soc Media Soc*, *8*(3), 20563051221122463. <https://doi.org/10.1177/20563051221122463>

Hong, Y., Wan, M., & Li, Z. (2021). Understanding the Health Information Sharing Behavior of Social Media Users. *Journal of Organizational and End User Computing*, *33*(5), 180-203. <https://doi.org/https://doi.org/10.4018/JOEUC.20210901.oa9>

Hossain Parash, M., Suki, N. M., Shimmi, S. C., Hossain, A., & Murthy, K. D. (2020). Examining students' intention to perform voluntary blood donation using a theory of planned behaviour: A structural equation modelling approach. *Transfus Clin Biol*, *27*(2), 70-77. <https://doi.org/10.1016/j.tracli.2020.02.002>

Ika Tamrin, S., Norman, A. A., & Hamid, S. (2021). Intention to share: the relationship between cybersecurity behaviour and sharing specific content in Facebook. *Information Research: an international electronic journal*, *26*(1). <https://doi.org/10.47989/irpaper894>

Islam, T., Mahmood, K., Sadiq, M., Usman, B., & Yousaf, S. (2020). Understanding Knowledgeable Workers’ Behavior Toward COVID-19 Information Sharing Through WhatsApp in Pakistan. *Frontiers in psychology*, *11*, 1-11. <https://doi.org/10.3389/fpsyg.2020.572526>

Joyner, L., Buchanan, T., & Yetkili, O. (2023). Moral leniency towards belief-consistent disinformation may help explain its spread on social media. *PLoS One*, *18*(3), e0281777. <https://doi.org/10.1371/journal.pone.0281777>

Kim, H. K., & Lai, C.-H. (2020). Seeking and Sharing Information about Transboundary Air Pollution in Singapore: Effects of Own and Others’ Information Insufficiency. *Environmental Communication*, *14*(1), 68-81. <https://doi.org/10.1080/17524032.2019.1597751>

Kim, H. S., Cho, K. M., & Kim, M. (2019). Information-Sharing Behaviors Among Sports Fans Using #Hashtags. *Communication & Sport*, *9*(4), 646-669. <https://doi.org/10.1177/2167479519878466>

Kim, J., Namkoong, K., & Chen, J. (2020). Predictors of Online News-Sharing Intention in the U.S and South Korea: An Application of the Theory of Reasoned Action. *Communication Studies*, *71*(2), 315-331. <https://doi.org/10.1080/10510974.2020.1726427>

Kim, S.-E., Kim, H. L., & Lee, S. (2021). How event information is trusted and shared on social media: a uses and gratification perspective. *Journal of Travel & Tourism Marketing*, *38*(5), 444-460. <https://doi.org/10.1080/10548408.2021.1943600>

Le, X. C. (2022). Propagation of information-sharing in social media: the perspective of intrinsic and extrinsic cues. *VINE Journal of Information and Knowledge Management Systems*, *54*(5), 973-989. <https://doi.org/10.1108/vjikms-01-2022-0006>

Lee, J. (2020). When we seek and share information about mental illness: The impact of threat appraisal, negative emotions, and efficacy. *The Social Science Journal*, *59*(4), 559-573. <https://doi.org/10.1080/03623319.2020.1727248>

Li, Y., Hu, Y., & Yang, S. (2022). Understanding social media users' engagement intention toward emergency information: the role of experience and information usefulness in a reciprocity framework. *Information Technology & People*, *36*(4), 1459-1483. <https://doi.org/10.1108/itp-10-2021-0753>

Lin, X., & Wang, X. (2020). Examining gender differences in people’s information-sharing decisions on social networking sites. *International Journal of Information Management*, *50*, 45-56. <https://doi.org/10.1016/j.ijinfomgt.2019.05.004>

Liu, Q., Su, F., Mu, A., & Wu, X. (2024). Understanding Social Media Information Sharing in Individuals with Depression: Insights from the Elaboration Likelihood Model and Schema Activation Theory. *Psychol Res Behav Manag*, *17*, 1587-1609. <https://doi.org/10.2147/PRBM.S450934>

Lu, H., & Yuan, S. (2021). What motivates information sharing about disaster victims on social media? Exploring the role of compassion, sadness, expectancy violation, and enjoyment. *International Journal of Disaster Risk Reduction*, *63*. <https://doi.org/10.1016/j.ijdrr.2021.102431>

Lu, L., Liu, J., Yuan, Y. C., Burns, K. S., Lu, E., & Li, D. (2021). Source Trust and COVID-19 Information Sharing: The Mediating Roles of Emotions and Beliefs About Sharing. *Health Educ Behav*, *48*(2), 132-139. <https://doi.org/10.1177/1090198120984760>

Lu, X., Vijaykumar, S., Jin, Y., & Rogerson, D. (2022). Think before you Share: Beliefs and emotions that shaped COVID-19 (Mis)information vetting and sharing intentions among WhatsApp users in the United Kingdom. *Telematics and Informatics*, *67*. <https://doi.org/10.1016/j.tele.2021.101750>

Luo, C., Li, H., Luo, X. R., & Cui, X. (2023). Exploring the effects of sense of membership on information sharing in virtual communities. *Journal of Electronic Commerce Research*, *24*(2), 107-126.

Lv, H., Cao, X., Chen, S., & Liu, L. (2022). Public and Private Information Sharing under "New Normal" of COVID-19: Understanding the Roles of Habit and Outcome Expectation. *Int J Environ Res Public Health*, *19*(9). <https://doi.org/10.3390/ijerph19095552>

Lyngdoh, T., El‐Manstrly, D., & Jeesha, K. (2022). Social isolation and social anxiety as drivers of generation Z's willingness to share personal information on social media. *Psychology & Marketing*, *40*(1), 5-26. <https://doi.org/10.1002/mar.21744>

Malik, A., Islam, T., Ahmad, M., & Mahmood, K. (2022). Health information seeking and sharing behavior of young adults on social media in Pakistan. *Journal of Librarianship and Information Science*, *55*(3), 579-595. <https://doi.org/10.1177/09610006221090228>

Malik, A., Mahmood, K., & Islam, T. (2021). Understanding the Facebook Users' Behavior towards COVID-19 Information Sharing by Integrating the Theory of Planned Behavior and Gratifications. *Information Development*, *39*(4), 750-763. <https://doi.org/10.1177/02666669211049383>

Marler, L. E., Cox, S. S., Simmering, M. J., Rogers, B. L., & Matherne, C. F. (2021). Can do and reason to: when are proactive employees willing to share negative information? *International Journal of Organizational Analysis*, *31*(3), 646-660. <https://doi.org/10.1108/ijoa-02-2021-2616>

Miri, A., Karimi-Shahanjarin, A., Afshari, M., Tapak, L., & Bashirian, S. (2024). The impact of emotional vs rational message framing on social media users' detection and sharing of misinformation: an experimental study. *Journal of Information, Communication and Ethics in Society*, *22*(3), 321-330. <https://doi.org/10.1108/jices-10-2023-0124>

Noh, S. (2021). Why do We Share Information? Explaining Information Sharing Behavior through a New Conceptual Model between Sharer to Receiver within SNS. *Asia pacific journal of information systems*, *31*(3), 392-414. <https://doi.org/https://doi.org/10.14329/apjis.2021.31.3.392>

Paek, H. J., Oh, H. J., & Hove, T. (2024). Differential effects of digital media platforms on climate change risk information-sharing intention: A moderated mediation model. *Risk Anal*, *44*(8), 1828-1838. <https://doi.org/10.1111/risa.14270>

Pei, X., Huang, Z., Yang, Y., & Bestoon, O. (2022). Research on user donation and information sharing in textile crowdfunding. *Industria Textila*, *73*(06), 671-679. <https://doi.org/10.35530/it.073.06.202195>

Pennycook, G., McPhetres, J., Zhang, Y., Lu, J. G., & Rand, D. G. (2020). Fighting COVID-19 Misinformation on Social Media: Experimental Evidence for a Scalable Accuracy-Nudge Intervention. *Psychol Sci*, *31*(7), 770-780. <https://doi.org/10.1177/0956797620939054>

Saling, L. L., Phillips, J. G., & Cohen, D. B. (2024). Accuracy-sensitisation promotes the sharing of pro- (but not anti-) vaccine information. *Psychol Health*, *39*(11), 1540-1554. <https://doi.org/10.1080/08870446.2023.2179053>

Shang, L., Zhou, J., & Zuo, M. (2020). Understanding older adults' intention to share health information on social media: the role of health belief and information processing. *Internet Research*, *31*(1), 100-122. <https://doi.org/10.1108/intr-12-2019-0512>

Shi, R., Jia, X., Hu, Y., & Wang, H. (2024). The media risk of infodemic in public health emergencies: Consequences and mitigation approaches. *PLoS One*, *19*(9), e0308080. <https://doi.org/10.1371/journal.pone.0308080>

Shree, T. (2024). How do users select the content they share on social media: flow theory perspective. *Online Information Review*. <https://doi.org/10.1108/oir-01-2022-0021>

So, J., Shim, M., & Song, H. (2023). Diffusion of COVID-19 misinformation: Mechanisms for threat- and efficacy-related misinformation diffusion. *Computers in Human Behavior*, *149*. <https://doi.org/10.1016/j.chb.2023.107967>

Soltani-Nejad, N., Mirezati, S. Z., & Saberi, M. K. (2020). Predicting intention to share information on commercial websites based on personality traits. *The Bottom Line*, *33*(3), 251-261. <https://doi.org/10.1108/bl-02-2020-0018>

Suárez Vázquez, A., & Chica Serrano, M. (2021). Others' fortune in online vs offline settings: how envy affects people's intention to share information. *Internet Research*, *31*(5), 1641-1655. <https://doi.org/10.1108/intr-10-2019-0412>

Sun, L., Wang, T., & Guan, F. (2021). How the strength of social ties influences users’ information sharing and purchase intentions. *Current Psychology*, *42*(9), 7712-7726. <https://doi.org/10.1007/s12144-021-02102-x>

Tang, Y., Luo, C., & Su, Y. (2024). Understanding health misinformation sharing among the middle-aged or above in China: roles of social media health information seeking, misperceptions and information processing predispositions. *Online Information Review*, *48*(2), 314-333. <https://doi.org/10.1108/OIR-04-2023-0157>

Toh, Z., & Lee, D. S. (2022). Is that Insta worthy? Predicting content sharing behavior on social media through interpersonal goals. *Cyberpsychology: Journal of Psychosocial Research on Cyberspace*, *16*(4). <https://doi.org/10.5817/cp2022-4-5>

Tseng, S.-M. (2023). Exploring the Information-Sharing Intention on Social Networking Sites. *Asia pacific journal of information systems*, *33*(2), 367-388. <https://doi.org/10.14329/apjis.2023.33.2.367>

Wicks, S. G., Hales, A. H., & Hennes, E. P. (2023). Does disseminating (mis)information restore social connection during a global pandemic? *Social and Personality Psychology Compass*, *17*(10). <https://doi.org/10.1111/spc3.12825>

Wu, X., & Kuang, W. (2021). Exploring Influence Factors of WeChat Users’ Health Information Sharing Behavior: Based on an Integrated Model of TPB, UGT and SCT. *International Journal of Human–Computer Interaction*, *37*(13), 1243-1255. <https://doi.org/10.1080/10447318.2021.1876358>

Xiang, M., Guan, T., Lin, M., Xie, Y., Luo, X., Han, M., & Lv, K. (2023). Configuration Path Study of Influencing Factors on Health Information-Sharing Behavior among Users of Online Health Communities: Based on SEM and fsQCA Methods. *Healthcare*, *11*(12), 1789. <https://doi.org/10.3390/healthcare11121789>

Xue, X., Ma, H., Zhao, Y. C., Zhu, Q., & Song, S. (2024). Mitigating the influence of message features on health misinformation sharing intention in social media: Experimental evidence for accuracy-nudge intervention. *Soc Sci Med*, *356*, 117136. <https://doi.org/10.1016/j.socscimed.2024.117136>

Yang, F., & Overton, H. (2022). What If Unmotivated Is More Dangerous? The Motivation-Contingent Effectiveness of Misinformation Correction on Social Media. *International Journal of Communication*, *16*, 27.

Yossatorn, Y., Binali, T., Weng, C., & Chu, R. J. (2023). Investigating the Relationships Among LINE Users’ Concerns, Motivations for Information Sharing Intention and Information Sharing Behavior. *Sage Open*, *13*(3). <https://doi.org/10.1177/21582440231192951>

Zhang, J., Xie, C., Chen, Y., & Lin, Z. (2023). How risk messages influence tourist processing and sharing: The role of emojis. *Journal of Hospitality and Tourism Management*, *56*, 454-468. <https://doi.org/10.1016/j.jhtm.2023.08.001>

Zhang, M., Li, Y., Gu, R., & Luo, C. (2021). What type of purchase do you prefer to share on social networking sites: Experiential or material? *Journal of Retailing and Consumer Services*, *58*. <https://doi.org/10.1016/j.jretconser.2020.102342>

Zhang, M., Lin, W., Ma, Z., Yang, J., & Zhang, Y. (2021). Users’ health information sharing intention in strong ties social media: context of emerging markets. *Library Hi Tech*, *41*(3), 853-876. <https://doi.org/10.1108/lht-02-2020-0024>

Zhang, Z., & Cheng, Z. (2024). Users' unverified information-sharing behavior on social media: The role of reasoned and social reactive pathways. *Acta Psychol (Amst)*, *245*, 104215. <https://doi.org/10.1016/j.actpsy.2024.104215>

Zhao, H., Fu, S., & Chen, X. (2020). Promoting users' intention to share online health articles on social media: The role of confirmation bias. *Inf Process Manag*, *57*(6), 102354. <https://doi.org/10.1016/j.ipm.2020.102354>
